# Cooperative behavior in the workplace: Empirical evidence from the agent-deed-consequences model of moral judgment

**DOI:** 10.3389/fpsyg.2022.1064442

**Published:** 2023-01-09

**Authors:** Sebastian Sattler, Veljko Dubljević, Eric Racine

**Affiliations:** ^1^Faculty of Sociology, Bielefeld University, Bielefeld, Germany; ^2^Institute of Sociology and Social Psychology, University of Cologne, Cologne, Germany; ^3^Pragmatic Health Ethics Research Unit, Institut de recherches cliniques de Montréal, Montréal, QC, Canada; ^4^Department of Philosophy and Religious Studies, North Carolina State University, Raleigh, NC, United States; ^5^Department of Medicine, Université de Montréal, Montréal, QC, Canada; ^6^Department of Preventive and Social Medicine, Université de Montréal, Montréal, QC, Canada; ^7^Department of Neurology and Neurosurgery, Biomedical Ethics Unit, and Experimental Medicine, McGill University, Montréal, QC, Canada

**Keywords:** agent-deed-consequences model, moral judgment, cooperative behavior, teamwork, drug misuse, survey experiment, vignette, cognitive enhancement

## Abstract

**Introduction:**

Moral judgment is of critical importance in the work context because of its implicit or explicit omnipresence in a wide range of work-place practices. The moral aspects of actual behaviors, intentions, and consequences represent areas of deep preoccupation, as exemplified in current corporate social responsibility programs, yet there remain ongoing debates on the best understanding of how such aspects of morality (behaviors, intentions, and consequences) interact. The ADC Model of moral judgment integrates the theoretical insights of three major moral theories (virtue ethics, deontology, and consequentialism) into a single model, which explains how moral judgment occurs in parallel evaluation processes of three different components: the character of a person (Agent-component); their actions (Deed-component); and the consequences brought about in the situation (Consequences-component). The model offers the possibility of overcoming difficulties encountered by single or dual-component theories.

**Methods:**

We designed a 2 × 2 × 2-between-subjects design vignette experiment with a Germany-wide sample of employed respondents (*N* = 1,349) to test this model.

**Results:**

Results showed that the Deed-component affects willingness to cooperate in the work context, which is mediated *via* moral judgments. These effects also varied depending on the levels of the Agent- and Consequences-component.

**Discussion:**

Thereby, the results exemplify the usefulness of the ADC Model in the work context by showing how the distinct components of morality affect moral judgment.

## 1. Introduction

Moral judgment is of critical importance in cooperative behavior in the work context because of its implicit omnipresence in a wide range of workplace practices (e.g., as part of everyday cooperative behavior between colleagues; due to concerns about scandals or backlash), but also its explicit existence (e.g., in codes of ethics) (Lee et al., 2019; e.g., Curtis et al., 2021). The moral aspects of how people act (i.e., their behaviors/deeds), their character traits and intentions, and the consequences, all represent (or should represent) areas of deep preoccupation. The cost of neglecting them has repeatedly shown to profoundly impact organizational culture and practices (DiFonzo et al., 2020). Yet, there remain ongoing debates on the best understanding of how such aspects of morality interact and, thus, models of intervention to tackle immoral behavior in cooperative work contexts reflect considerable diversity. This diversity can be traced back to theoretical and methodological divergence which guide interventions in organizational ethical culture. While deontology-oriented theories point to knowledge of principles enshrined in codes of ethics; consequence-oriented theories stress the importance of moral conduct, including sanctioning immoral behavior and incentivizing moral behavior (Sager, 2017; Salazar, 2017). Current debates about the actual worth and scientific rigor of widespread social responsibility programs (Craze, 2020; Noble and Dubljević, 2022) raise such questions about the kind of theory and goals which should orient interventions at workplaces. At the same time, narrow orientations on fitting with rules (as with the Volkswagen scandal; Hotten, 2015) or meeting certain profit goals (as with Wells Fargo; Tayan, 2019) provide ample evidence that a more holistic approach is urgently needed.

In cooperative working contexts (e.g., behavioral ethics; Bazerman and Tenbrunsel, 2011) and beyond, moral judgment is increasingly understood as relying on heuristic-based evaluations that may occur with or without limited conscious deliberation (Reynolds, 2006; Kahneman, 2013). Although there are multiple models of moral judgment stemming from empirical moral psychology (Sunstein, 2005; Mikhail, 2007), a new model—the Agent-Deed-Consequences (ADC) model—is reflective of three major ethical theories (Dubljević, 2021). This model integrates assumptions of three distinct ethical theories, whereby each theory concentrates on specific aspects of moral judgment: virtue ethics, which focuses on the intentions and character of a person involved in the situation; deontology, which focuses on the analysis of certain actions that are either prohibited or required as a duty; and consequentialism, which focuses on the balance of harms and gains resulting from the situation (Dubljević et al., 2021). Research has shown that the moral judgments of individuals that do not have explicit knowledge of ethics correspond to the moral precepts implied in moral theories (Dubljević et al., 2018; Cacace et al., 2022). This validates the psychological reality and usefulness of these major theories. However, traditionally, these single-component theories of moral judgment have struggled to take into account and compute the three possibly concurrent precepts (Dewey, 1930; Dubljević and Racine, 2014).

In response, the ADC Model takes into account all three of these different components of moral judgment and offers a workable plural model of such judgment. It explains—building on previous foundational work on moral heuristics (Sunstein, 2005) and Universal Moral Grammar (Mikhail, 2007)—that moral judgment is based on simultaneous evaluations of these three different components of a situation: the character of a person (the Agent-component, A); their actions (the Deed-component, D); and the consequences brought about in a given situation (the Consequences-component, C). Basic and heuristic-like processing of moral intuitions can be computed within a process of quick moral judgment, required by social cooperation (Boyd and Richerson, 2009). According to the integrative ADC Model, the moral evaluation of a situation happens through a heuristic processing of cues. These psychological cues, or cognitive short-hand, substitute the overall moral judgment with more accessible information in the form of these three distinct computations which are combined to form the moral judgments. For example, if the Agent, Deed, and Consequences are all positively charged (prima facie perception of good), the observer will evaluate the situation as morally acceptable or positive. For example, if a courageous woman [Agent (+)] jumps into a pond to save a drowning baby [Deed (+)] and everyone survives and is healthy and happy [Consequence (+)], the moral judgment of the situation will be positive [Moral judgment (+)]. Conversely, if the Agent, Deed, and Consequences are all bad, the situation will be judged to be morally unacceptable or negative. For example, if a sadist [Agent (-)] attacks a woman [Deed (-)] and she dies [Consequence (-)], the moral judgment will be clearly negative [Moral Judgment (-)]. An important question, however, is how moral judgments are made when the valence of these three components does not align. The ADC Model proposes to frame such situations with contrasting moral aspects as simple computations. For instance, if the character and intentions of a person are good, and the Deed is good, individuals may be more likely to accept or excuse bad Consequences ([Agent (+)], [Deed (+)], and [Consequence (-)] may result in [Moral judgment (+)]). For example, if a courageous woman [Agent (+)] jumped into a pond to try saving a drowning baby [Deed (+)], but the baby still drowns [Consequence (-)], impartial observers are still likely to praise the Agent and the Deed, regardless of the Consequences. Similarly, if a courageous woman [Agent (+)] attacks another woman [Deed (-)] who is trying to drown a baby and succeeds in saving the baby's life [Consequence (+)], impartial observers would likely excuse the norm violation, leading to a positive evaluation of the situation [Moral judgment (+)]. The interesting question arises when asking whether similar norm violations can be excused by intentions and consequences that are less dramatic.

In principle, this kind of parallel processing and moral judgment computing should apply across the board: with both dramatic/“high-stakes” (i.e., involving possible death) or mundane/“low-stakes” situations (i.e., involving everyday norm violations, such as lying). However, prior work (Dubljević et al., 2018) has noted that lying, as a negative Deed, seems to have a greater effect than other aspects of the situation (i.e., Agent and Consequences) in moral judgments of “low-stakes” situations. Other bad Deeds and norm violations need to be explored in multiple contexts in order to draw firmer conclusions. To better understand these evaluative processes, it is also necessary to examine whether and how the three components (Agent, Deed, and Consequence) interact with one another, as well as how they may affect behavioral tendencies (e.g., willingness to cooperate with someone). Namely, it is important to understand if the computation is carried out according to a function of basic summation and if the weight attributed to different components of the situation are somehow calibrated as part of our situational understanding of human realities where the different components would change weight depending on the situation (e.g., Mischel, 1977). It can be argued that congruence between the Deed and the Agent's intention to engage in a Deed reinforce each other, as does the valence of the Deed and its Consequences. For example, a positive Agent's intention, together with a positive Deed, may signal that a good behavior is not a singularity but part of a stable disposition (Dubljević et al., 2018). Such congruence aligns with the argument that moral integrity describes consistent actions and a person's character (Jacobs, 2004). Similarly, consistently performing good Deeds resulting in good Consequences, signals congruence as well.

This model advantageously prevents unreasonable conclusions that stem from single-component theories (e.g., one should not lie even to save all humanity). It also provides a long sought-after three-pronged integrative account that helps clarify the multifaceted nature of moral judgment (Dewey, 1930). The ADC Model suggests that, while this process is mostly unconscious, conscious processes might monitor and correct moral judgments. This is in line with contemporary findings of the duality of cognitive systems pioneered in economics (Tversky and Kahneman, 1974, 1981; Kahneman, 2013). Also, the model provides guidance when precepts from single-component moral theories lead to counter-intuitive positions (e.g., lying to a serial killer may be viewed as deontologically wrong, but still morally acceptable).

There is already partial and indirect support for a three-component model such as the ADC Model in the literature on cooperative behavior in the workplace. For instance, Arikan (2020) found that opportunism judgments (a moral judgment of an unethical act in the workplace) are influenced by (a) the type of the behavior (or “Deed” in our nomenclature), (b) the type of the causal account provided for the behavior (or the connection between “Agent” and “Deed” in our nomenclature), (c) the perceived type of the exchange (or “Consequence” in our nomenclature), and (d) the personality traits of the actor (or “Agent” in our nomenclature). For example, perceiving that a transgressor experiences remorse for their organizational crimes (i.e., that they are not entirely a bad “Agent”) can deter people from whistleblowing. The effect of remorse is particularly strong if the transgressor is part of a cohesive and homogenous work group, thus signaling the role of moral norms about deeds (Khan and Howe, 2021). Similar findings are reported in other studies as well. Reduced intentionality on the part of the agent greatly impacts the moral judgment of co-workers and subsequent punishment following a transgression (Zhang et al., 2019). These and other findings (Kim et al., 2017; Brown-Liburd et al., 2018; e.g., Blay et al., 2019; Ellemers et al., 2019; Wang et al., 2019; Keck et al., 2020; Jain and Lee, 2022) are evidence that moral judgment cannot be simply understood following single-component or even two-component theories. Thus, it would be beneficial for all three sources of moral intuitions to be envisioned as part of an integrative computing process.

In order to increase our understanding of moral judgment and its underlying parallel processes in cooperative behavior in the workplace, we set out to examine whether different Deeds have the strongest effects (Dubljević, 2021) and how the effects of the Agent, Deed, and Consequence components interact. We extend the investigation of the ADC Model by testing further consequences of the ADC components, that is, whether they also affect willingness to cooperate *via* moral judgment (Tomasello and Vaish, 2013). Investigating such processes is of crucial importance, for example, because much work happens in groups and increasing our understanding of conditions for cooperative behavior (Hackman and Morris, 1975; Bond and Titus, 1983; Karau and Williams, 1993), can help increase productivity and inform ethical training in many types of organizations (Sturm, 2017; e.g., Martineau et al., 2020). Given that moral judgments are known to correlate with intended or actual behavior (Ajzen, 1991; Tittle et al., 2010; Sattler et al., 2021; Huber et al., 2022), it can be reasonably assumed that such judgments are antecedents to the behavioral willingness to conduct a certain behavior. Acting against one's moral concerns can lead to negative emotions or more generally psychological costs, while behavior aligning with morality should lead to the opposite, i.e., intrinsic benefits (Coleman, 1994; Posner and Rasmusen, 1999; Opp, 2013). Moreover, moral evaluations can also serve as definitions or frames of the situation and thereby guide decision-making consciously or unconsciously (Kroneberg, 2014; Sattler et al., 2021). In addition, intentions can be seen as proximal antecedents of future behavior (Ajzen, 1991; Gibbons et al., 1998). They capture motivational factors to perform a certain behavior (Grasmick and Bursik, 1990; Ajzen, 1991; Gibbons et al., 1998). While the focus of this study is to investigate moral judgments as a mediator for willingness to cooperate, it should be acknowledged that this mediation is only partial, meaning that the ADC components may also affect this willingness *via* other mediators such as personal monetary and non-monetary consequences for the cooperating partner (e.g., negative Consequences in one interaction may reduce willingness to engage in further cooperation) or effects of trust (e.g., a bad intent of the Agent may decrease trust and in consequence willingness to cooperate), which could explain remaining direct effects of the components (Mo and Shi, 2017; Khan and Howe, 2021).

## 2. The current study

Based on the reasoning above, this study serves several goals. First, we want to re-test the main hypotheses of the ADC Model:

1. Positive Agent intentions result in more positive moral judgments as compared to negative Agent intentions.
2. Positive Deeds result in more positive moral judgments as compared to negative Deeds.
3. Positive Consequences result in more positive moral judgments as compared to negative Consequences.

Second, before testing hypotheses on the more complex mediating and moderating relations between the ADC components, moral judgments, and willingness to cooperate, we want to explore whether the ADC components affect this willingness to cooperate:

4. Positive Agent intentions result in a higher willingness to cooperate as compared to negative Agent intentions.
5. Positive Deeds result in a higher willingness to cooperate as compared to negative Deeds.
6. Positive Consequences result in a higher willingness to cooperate as compared to negative Consequences.

**Figure 1:**
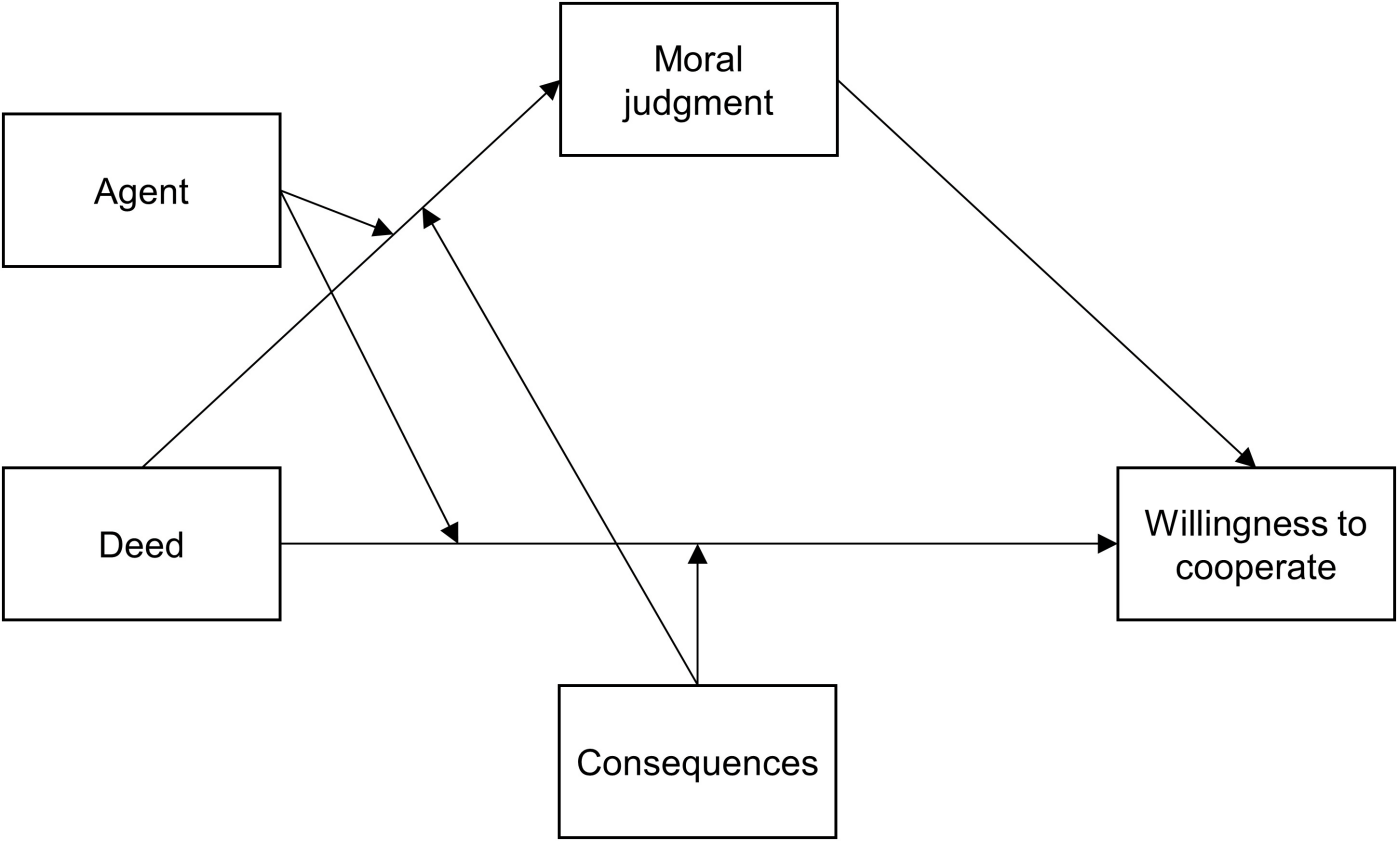
Moderated mediation model.

Third, based upon assumptions from the ADC Model and research on the relation between moral judgment and intended behavior, we want to test the following hypotheses (see Figure 1 as a graphical representation of the proposed model):

7. Deed effects on willingness are partially mediated by moral judgments, i.e., the effect of a positive Deed on willingness is *via* more positive moral judgments.
8. Positive Agent intentions increase the positive effect of a positive Deed on moral judgments, thereby, the Agent's intention moderates the mediation effect between Deed and willingness *via* moral judgments.
9. Positive Consequences increase the positive effect of a positive Deed on moral judgments, thereby, the Consequences moderate the mediation effect between Deed and willingness *via* moral judgments.
10. The remaining direct effect of the Deed on willingness is stronger if the Agent's intention is positive (rather than negative).
11. The remaining direct effect of the Deed on willingness is stronger if the Consequence is positive (rather than negative).

The current study builds from previous investigation of the ADC Model to explore cooperative behavior in the workplace and to validate and replicate previous findings therein (Dubljević et al., 2018) while using a larger, more representative and heterogeneous sample. We also want to extend beyond previous findings on this model to examine how the ADC components indirectly (*via* moral judgment) and directly affect willingness to cooperate in occupational contexts. Thus, we want to explore whether the ADC components also have relevance for decision-making in choosing a certain work-relevant actions or asking for medical services. The large sample of representatively selected employed adults in Germany (rather than using student samples or frequently used crowd-sourced samples, e.g., US American MTurk) allows us to test the ADC Model in another cultural context and with a more heterogeneous sample. The use of our experimental design in connection with moderated mediation models allows for a causally-oriented test of the ADC Model and its consequences for behavioral willingness.

We chose to investigate the ADC Model in the context of drug misuse in the workplace, which has been recently discussed as a severe problem because of the health risks to employees and employers, which could result in absenteeism, work-place accidents, and several other important problems such as the (indirect) pressure from employers and peers to use certain drugs for better job performance (d'Angelo et al., 2017; Leon et al., 2019; Dubljević et al., 2020; Huber et al., 2022). Drug misuse can therefore also bear profound societal and economic costs for health insurance and employers. Studies suggest that prescription and illegal drugs are used to deal with work stress or to enhance cognitive performance in the job (Frone, 2008; Maier et al., 2018; Baum et al., 2021; Sattler and von dem Knesebeck, 2022). For example, a study in 15 western countries found the United States to rank among the countries with the highest self-reported 12-month prevalence for illegal stimulant use (e.g., cocaine, amphetamine, or methamphetamine) with 14.7% for increasing cognitive performance at work or for studying without medical indication (Maier et al., 2018). Some scholars assumed that the public would preferentially fly with airlines or go to hospitals where drugs are used non-medically to increase cognitive performance of their employees (such as alertness) (Chatterjee, 2004; Bostrom and Sandberg, 2009). Those willing to use drugs could possibly have an edge when being hired, resulting in competition that might pressure others to engage in using such substances and make such use a social obligation (Faulmüller et al., 2013; Dubljević et al., 2014; Jane and Vincent, 2017; Racine et al., 2021). Thus, applying the ADC model in the context of drug misuse will increase our understanding of how such debated behavior may affect workplace interactions and draw attention to actions to be undertaken. So, due to the individual and societal risks of drug use in the work context, prevention and interventions might not only inform individuals about these risks but also about whether such drug use would really help them in workplace interactions or rather, lead to potentially negative consequences such as rejection as a non-cooperating partner or reduced demand for a service.

## 3. Methods

### 3.1. Design and participants

We conducted a web-based vignette experiment for which we recruited 1,349 employed participants (46.85% females; mean age: 49.973; *SD* = 11.973) who completed the experiment. Participants were part of a nationwide sample of German-speaking residents in private households in Germany with a minimum age of 18 (which applies to about 95% of all households Statista, 2020). The sample was based on a representative panel of the German population (forsa.omninet) that was recruited *via* a multi-stage, random process using a telephone master sample of the Association of German Market and Social Research Institutes (Arbeitskreis Deutscher Markt- und Sozialforschungsinstitute e.V., ADM). Thereby, every household in Germany had the same statistical chance to participate (and infrequent Internet users were reached). Self-selection into the panel or respondents with multiple accounts were prevented. Our experiment was part of a larger study aiming for greater heterogeneity and a more representative set of participants compared to common student or crowd sourced samples. After providing informed consent, participants filled in the survey and were financially compensated for their participation. This study was approved by the ethics committee of the University of Erfurt (reference number: EV-20190917).

### 3.2. Materials and procedure

#### 3.2.1. Factorial survey with vignettes

For our experiment, we employed a factorial survey design with vignettes to combine the advantages of experiments, such as high internal validity and non-multicollinearity of the treatments, with those of survey research, such as external validity due to more representative samples than in-lab experiments (Jasso, 2006; Atzmüller and Steiner, 2010; Aguinis and Bradley, 2014). Vignettes are short descriptions of hypothetical and experimentally varied situations. They are useful when manipulations in the “real world” are challenging due to ethical or practical reasons (Rettinger and Kramer, 2009; Graeff et al., 2014). Moreover, vignettes can reduce socially desired responding (Alexander and Becker, 1978; Wason et al., 2002; Sauer et al., 2011). We used a between-subject design to avoid learning and contrast effects (Göritz and Weiss, 2014) and thus randomly assigned each respondent to one of the vignettes. Each vignette varied in three dimensions (Agent, Deed, and Consequences), resulting in a 2 × 2 × 2 experiment describing a situation concerning team work (Table 1). The scenario involved drug misuse in this occupational setting.

**Table 1:** Vignette scenario with three dimensions and two levels each.

There is a company which is in a difficult financial situation. So, their product range is supposed to be revised. The employees are supposed to develop new ideas in groups and present them at the end of the day. Alexandra is in one of the groups. She is known for being very [Agent (-): *lazy* | Agent (+): *dedicated*]. To prepare for this teamwork, she decides to [Deed-: *take a small dose of the illegal amphetamine “speed”* | Deed (+): *go over all relevant documents on customer preferences and market demands*]. Because of Alexandra's preliminary work, her group develops ideas which are a lot [Consequence (-): *worse* | Consequence (+): *better*] than those of the other groups.

Text in square brackets indicates the three experimentally varied vignette dimensions with negative and positive valence of Agent, Deed, and Consequence. In the survey, the text was neither bolded nor italicized.

#### 3.2.2. Moral judgment

After reading the scenario, participants were asked: “Considering all of the circumstances, how morally acceptable do you find what Alexandra did in this situation?” (Tannenbaum et al., 2011; Sattler et al., 2013; for similar measures, see Dubljević et al., 2018). Response options ranged from “not at all” [1] to “completely” [10].

#### 3.2.3. Behavioral willingness

Participants then indicated their willingness to cooperate with the Agent in the form of engaging in teamwork (“If you were in a situation in which teamwork were necessary, would you want to work with Alexandra?”). Response options again ranged from “not at all” [1] to “completely” [10]. Such measures have shown high correlations with behavior (Beck and Ajzen, 1991; Pogarsky, 2004).

#### 3.2.4. Pretesting

To evaluate and improve the comprehensibility and validity of the instructions and instruments, the vignettes, items, and instructions underwent cognitive pretests (*N* = 9) with the think-aloud technique and probing questions (Van Someren et al., 1994), and we conducted a quantitative pretest (*N* = 63). Based on the pretest, minimal changes (e.g., edits in the wording to increase understanding) were made to make the materials more suitable for the nationwide sample.

### 3.3. Statistical analysis

To examine bivariate treatment effects on moral judgment and willingness, we ran *t*-tests. To further test the model described in Figure 1, we used first-stage moderated mediation models with Model 10 of the SPSS macro PROCESS (Hayes, 2017). These models tested the impact of the Deed on the behavioral willingness through the mediator moral judgment and whether the Agent and the Consequence moderated the effects of the Deed on the mediator and the willingness (see Figure 1). To increase the accuracy of the indirect effects, we used percentile bootstrap confidence intervals (with *N* = 5,000 bootstrap samples) (MacKinnon et al., 2004; Hayes, 2017). Thereby, a CI that does not include zero indicates a statistically significant effect. We used heteroscedasticity consistent standard errors (*HC3*) (Hayes and Cai, 2007).

## 4. Results

**Figure 2:**
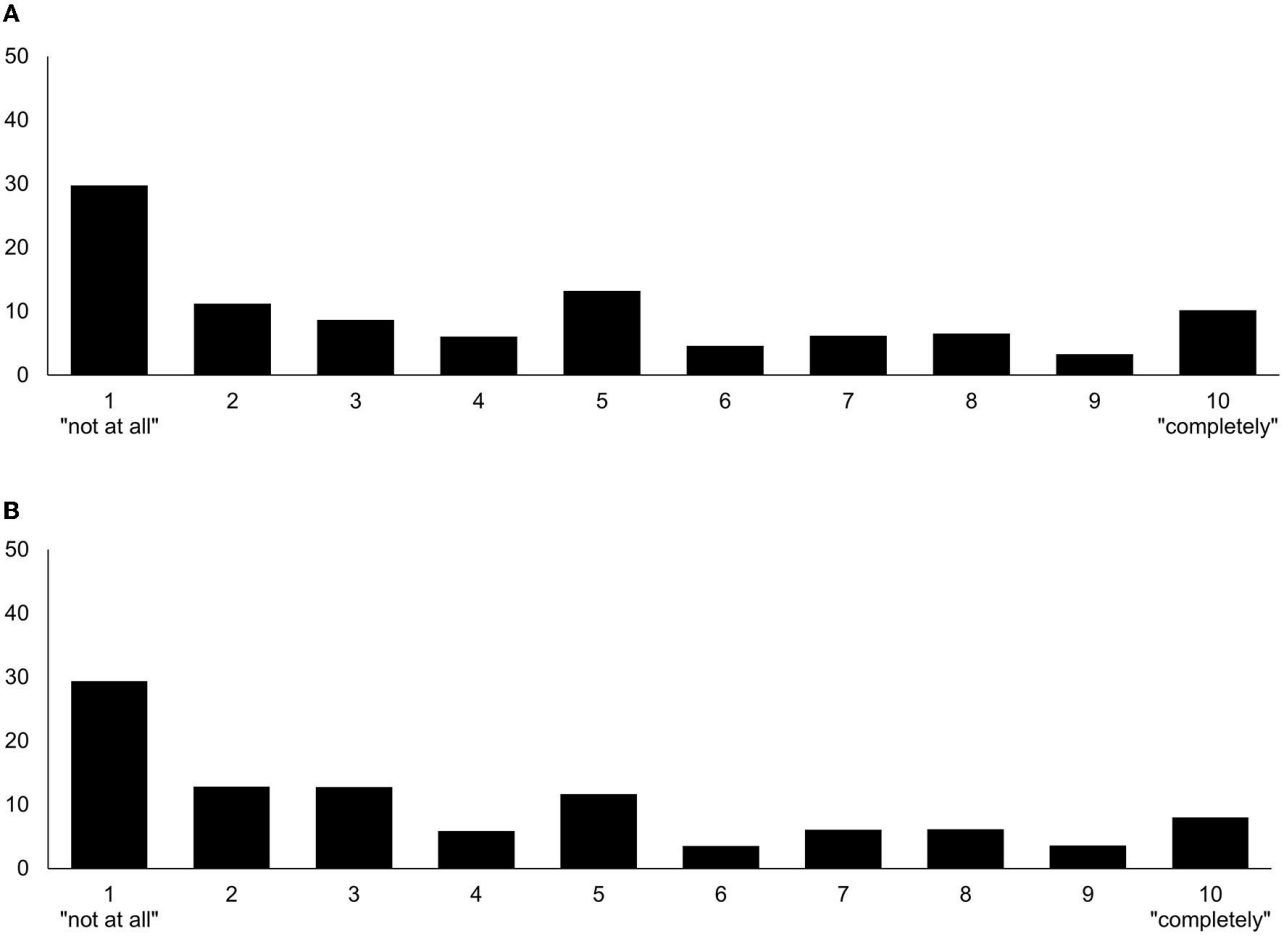
Distribution of answers (in %) of the moral judgment **(A)** and willingness **(B)** (*N* = 1,349).

Figure 2 shows that respondents on average considered the employee's behavior in the given situation, with its consequences, moderately morally acceptable (*M* = 4.25; *SD* = 3.10). The willingness to cooperate with the depicted employee was also moderate (*M* = 4.01; *SD* = 2.98). First, we tested whether the experimental manipulations of the three components (Agent, Deed, and Consequence) predicted an effect on moral judgment and intended behavior. Table 2 shows a statistically significantly more positive moral judgment if the team member engaged in a positive Deed as compared to a negative Deed (*p* < 0.001), if she had positive as compared to negative Agent intentions (*p* = 0.003), and if her action caused positive as compared to negative Consequences (*p* < 0.001). Similarly, the willingness to co-work with the team member in the future was stronger if the team member engaged in a positive Deed as compared to a negative Deed (*p* < 0.001), if she had positive as compared to negative intentions (*p* < 0.001), and if her action caused positive as compared to negative Consequences (*p* < 0.001). The results showed that moral judgment and willingness strongly correlate (*r* = 0.703, *p* < 0.001). As part of the moderated mediation analysis, the mediator variable model (Table 3) with moral judgment as the outcome showed a conditional main effect of the Deed (*p* < 0.001) on moral judgment. The situation was judged more positively if the team member engaged in a positive as compared to a negative Deed, even in the case the team member had bad intentions (A-) or her Deed had negative Consequences. Both statistically insignificant conditional main effects of the Agent (*p* = 0.866) and the Consequences (*p* = 0.439) suggested that these components did not provoke different judgments in the case of a negative Deed.

**Table 2:** Bivariate treatment effects (*N* = 1,349).

	**Negative (-)**		**Positive (**+**)**			
	**M**	**SD**	**M**	**SD**	***t*-value**	**Cohen's *d***
**Moral judgment**						
Agent	3.99	3.010	4.50	3.166	−2.994^**^	−0.163
Deed	2.68	2.388	5.82	2.931	−21.541^***^	−1.173
Consequence	2.67	3.610	4.88	3.338	−7.679^***^	−0.418
**Willingness**						
Agent	3.64	2.797	4.38	3.107	−4.565^***^	0.249
Deed	2.79	2.398	5.24	3.001	−16.580^***^	−0.903
Consequence	3.08	2.406	4.94	3.197	−12.100^***^	−0.659

** p < 0.01,

*** p < 0.001; M, Mean value; SD, Standard deviation.

**Table 3:** Mediator variable model of the conditional mediation model (*N* = 1,349).

	**Effect**	**SE**	**CI**
**Mediator variable models for the outcome moral judgment**			
Deed + (Ref. -)	1.506^***^	0.238	[1.039, 1.973]
Agent + (Ref. -)	0.031	0.184	[−0.331, 0.393]
Consequence + (Ref. -)	0.143	0.184	[−0.219, 0.504]
Deed^*^Agent	0.968^***^	0.274	[0.431, 1.505]
Deed^*^Consequence	2.295^***^	0.274	[1.758, 2.833]
Constant	2.592^***^	0.167	[2.264,2.921]
*R^2^* (*F-Test*)	0.347 (136.584^***^)		
**Conditional effect of Deed at different values of Agent and Consequence**			
Agent (-) & Consequence (-)	1.506^***^	0.238	[1.039, 1.973]
Agent (-) & Consequence (+)	3.801^***^	0.242	[3.326, 4.277]
Agent (+) & Consequence (-)	2.474^***^	0.232	[2.018, 2.930]
Agent (+) & Consequence (+)	4.769^***^	0.236	[4.306, 5.232]

CI, 95% confidence interval; SE, Standard error. ^*^p < 0.05, ^**^p < 0.01, ^***^p < 0.001.

**Figure 3:**
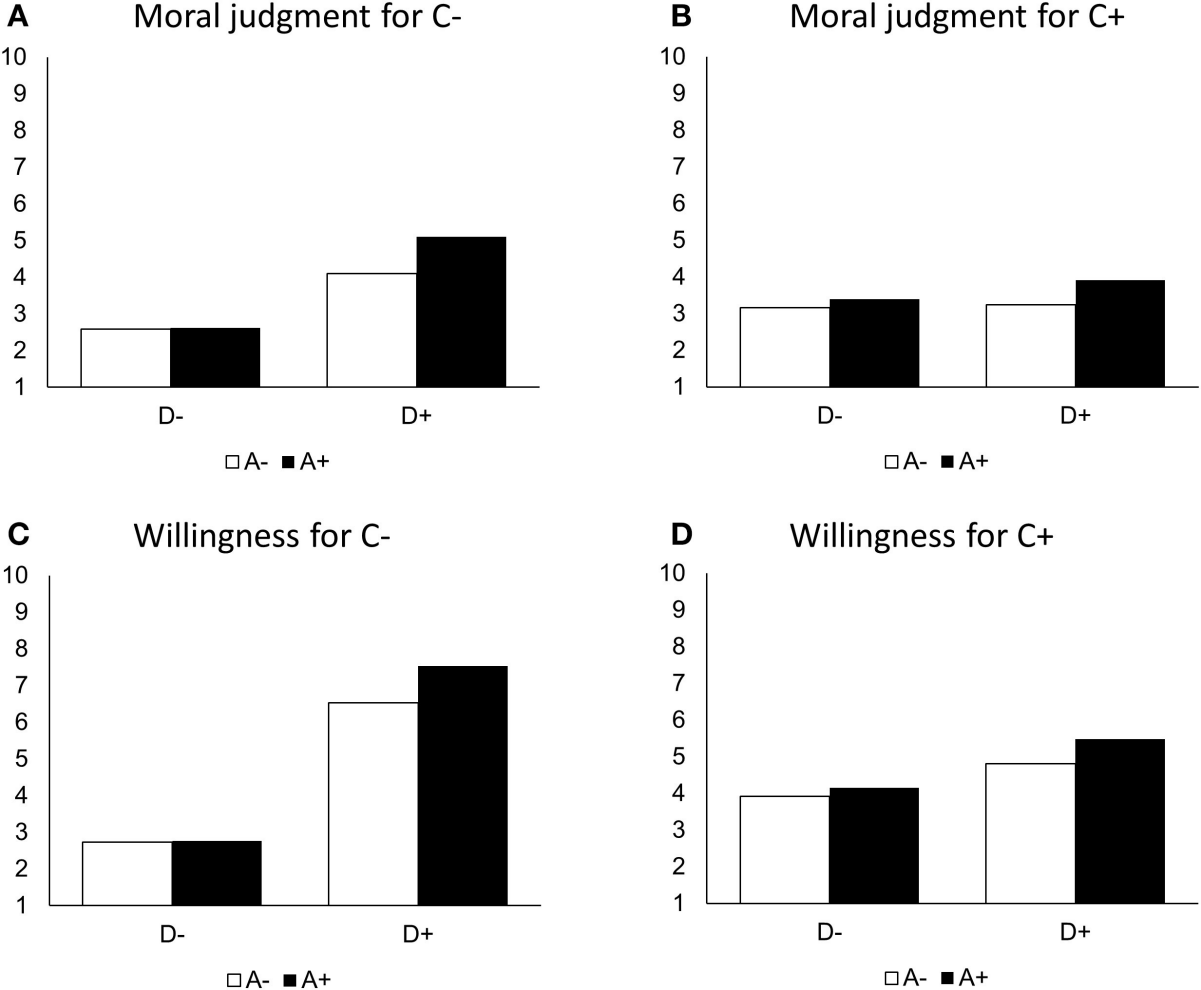
Predicted values for moral judgment **(A, B)** and willingness **(C, D)** (*N* = 1,349).

The statistically significant interaction effects between Deed and Agent (*p* < 0.001) and Deed and Consequences (*p* < 0.001) suggested that the positive effect of the Deed was reinforced if the team partner had positive rather than negative intentions and if there were positive rather than negative Consequences. The results also suggested that D had the strongest effect when A and C were both positive (*p* < 0.001) and the weakest effect when A and C were both negative (*p* < 0.001). See Figures 3A, B for a visualization of the findings.

The dependent variable model with willingness to cooperate with the team partner as the outcome (Table 4) revealed that moral judgment, as the suggested mediator (*p* < 0.001), exerted the expected positive effect on willingness to cooperate. This means that the more positively the situation was judged to be, the higher the willingness to cooperate with the team member in the future. Controlling for the mediator, the Deed had no statistically significant conditional main effect when Agent and Consequences were negative (*p* = 0.665). While the model showed no interaction effect between Deed and Agent (*p* = 0.135), it revealed a statistically significant interaction effect between Deed and Consequences (*p* < 0.001). This suggested that the Deed had a stronger effect if its Consequences were positive rather than negative. Although statistically insignificant when Agent and Consequences were both negative, the Deed had a positive conditional direct effect when either Agent and Consequences were positive, but especially when both were positive (Figures 3C, D).

**Table 4:** Dependent variable models of the conditional mediation models (*N* = 1,349).

	**Effect**	**SE**	**CI**
**Dependent variable: Willingness**			
Deed + (Ref. -)	0.087	0.202	[−0.308, 0.483]
Moral judgment	0.559^***^	0.029	[0.502, 0.615]
Agent + (Ref. -)	0.237	0.159	[−0.074, 0.548]
Consequence + (Ref. -)	0.758^***^	0.159	[0.446,1.071]
Deed^*^Agent	0.433	0.223	[−0.004, 0.871]
Deed^*^Consequence	0.803^***^	0.230	[0.352, 1.254]
Constant	0.786^***^	0.146	[0.498, 1.073]
*R^2^* (*F-Test*)	0.543 (313.132^***^)		
**Conditional direct effects of Deed**			
Agent (-) & Consequence (-)	0.087	0.202	[−0.308, 0.483]
Agent (-) & Consequence (+)	0.891^***^	0.226	[0.447, 1.334]
Agent (+) & Consequence (-)	0.520^*^	0.218	[0.094, 0.947]
Agent (+) & Consequence (+)	1.324^***^	0.243	[0.846, 1.801]
	**Effect**	**SE (Boot)**	**CI (Boot)**
**Conditional indirect effects of Deed** ***via*** **moral judgment**			
Agent (-) & Consequence (-)	0.842	0.144	[0.568, 1.131]
Agent (-) & Consequence (+)	2.124	0.167	[1.799, 2.458]
Agent (+) & Consequence (-)	1.383	0.155	[1.086, 1.688]
Agent (+) & Consequence (+)	2.665	0.183	[2.311,3.033]
	**Contrast**	**SE (Boot)**	**CI (Boot)**
**Pairwise contrasts between conditional indirect effects of moral judgment**			
Agent (-) & Consequence (+) vs. Agent (-) & Consequence (-)	1.283	0.154	[0.988, 1.592]
Agent (+) & Consequence (-) vs. Agent (-) & Consequence (-)	0.541	0.154	[0.244, 0.850]
Agent (+) & Consequence (+) vs. Agent (-) & Consequence (-)	1.824	0.224	[1.399, 2.264]
Agent (+) & Consequence (-) vs. Agent (-) & Consequence (+)	−0.742	0.213	[−1.161, −0.328]
Agent (+) & Consequence (+) vs. Agent (-) & Consequence (+)	0.541	0.154	[0.244, 0.850]
Agent (+) & Consequence (+) vs. Agent (+) & Consequence (-)	1.283	0.154	[0.988, 1.592]

^*^p < 0.05, ^**^p < 0.01, ^***^p < 0.001. CI, 95% confidence interval; SE, Standard error; Boot, Bootstrap sample size = 5,000.

Indicative for the moderated mediation are the indices of partial moderation that were statistically significant for Agent (*B* = 0.541; 95% CI [0.244, 0.850]) and Consequences (*B* = 1.283; 95% CI [0.988, 1.592]), denoting that the indirect effects of Agent and Consequences on willingness *via* moral judgment varied significantly across different values of Agent and Consequences. The conditional indirect effects showed the strongest effect of a positive Deed when both Agent and Consequences were positive, while the smallest effect existed when both were negative. A positive Deed appeared to exert stronger effects when a negative Agent was combined with a positive Consequence as compared to a positive Agent combined with a negative Consequence.

## 5. Discussion

This study set out to investigate a novel explanatory theory of moral judgment, the ADC Model of moral judgment (Dubljević and Racine, 2014) in the context of cooperative workplace behavior within a scenario-based experiment using a population-based sample. Beyond examining whether the ADC model can help understand moral judgment in this context, this study also tested whether the components of the model affect willingness to cooperate indirectly *via* moral judgment and whether remaining direct effects exist. We also tested whether the effect of the Deed on moral judgments and willingness to cooperate are moderated by the Agent's intentions and the Consequences of the Deed.

Our results show that the general hypotheses implied in the ADC Model were supported (Dubljević and Racine, 2014, 2017) in our teamwork scenario: A positive valence of the Agent (Hypothesis 1), Deed (Hypothesis 2), and Consequence (Hypothesis 3) in comparison to a negative valence of each component resulted in a more positive moral judgment. These results thereby confirm previous findings obtained in a different context and sample (Dubljević et al., 2018). Moreover, the Deed had the strongest effect on moral judgment, in line with previous findings (Reynolds, 2006; e.g., Dubljević et al., 2018). These findings imply that most single-component approaches are limited in both normative and descriptive senses. Most of the extant literature and current theories either favor one major moral theory or contrast two [e.g., dual process theory (e.g., Greene, 2007)], but our results further show significant limitations with this orientation. Moreover, we found that the ADC components also affected behavioral willingness, showing that they are not only relevant for moral judgments but have further impacts on interactions in professional contexts (providing support for Hypotheses 4 regarding the Agent, Hypothesis 5 regarding the Deed, and Hypothesis 6 regarding the Consequences). Our finding that the Deed revealed the strongest effect suggests that individuals are sensitive toward morally questionable behavior, while positive behavior results in more cooperative behavior or professional interactions (see below).

### 5.1. Interaction effects concerning moral judgment

For moral judgments, we found evidence for positive interaction effects between the Deed- and the Agent-component (replicating previous findings) and between the Deed- and the Consequences-component (supporting Hypotheses 8 and 9). Thus, a positive Agent intention to engage in a Deed and positive Consequences of the Deed (rather than negative ones) reinforced the positive effect of a positive Deed. This may confirm that when the Deed is congruent with other components of the model, the positive Deed “is not just a single instance of good behavior, but the agent's overall stable disposition,” which supports the common belief that good people act in good ways (Dubljević et al., 2018, p. 12). The importance of such congruence between intention and action has been described in moral theories, as in Kant's argument that a deed might only be good if it is motivated by good will (Humphrey, 2003). This also aligns with views that moral integrity can be understood in terms of the consistency of the agent's deeds with their character (Jacobs, 2004). The results also suggest that when the Deed was described as negative, both the Agent- and the Consequences-component appeared not to affect moral judgment. This suggests that the Deed is a key stimulus in moral judgment and that neither a positive Agent nor positive Consequences can change the moral judgment if the Deed is negative. Thus, the strong effect of a negative Deed results in disregard of the positive valence of the two other components. For instance, good consequences arising from the seemingly condemned use of drugs may be viewed as undeserved or incidental. Similarly, whatever the intention of the Agent, taking drugs may be viewed as morally tainting.

### 5.2. Moral judgment is a potential antecedent of behavioral willingness

In line with prior research on the relation between moral attitudes and behavioral willingness (Ajzen, 1991; Sattler et al., 2013; Wiegel et al., 2016; Bavarian et al., 2019; Huber et al., 2022), we found that more positive moral judgments resulted in higher willingness to cooperate. Such moral judgments might be antecedents when individuals unconsciously or consciously develop a willingness to conduct a certain behavior. The moral evaluation of the situation can guide the perception of behavioral options (Kroneberg, 2014; Sattler et al., 2021) and disregarding such moral beliefs would potentially lead to psychological costs created by morally problematic situations (Coleman, 1994; Posner and Rasmusen, 1999; Opp, 2013). Still, certain restrictions (e.g., money, time, skills, or opportunity) may prevent individuals from turning willingness into action. These findings may imply that human interaction, including (professional) cooperation and exchange, is profoundly moral in nature. While this has long been observed, our findings provide a nuanced view on how morality supports and nurtures cooperation. This raises important implications for organizational culture: instances of immoral behaviors, negative intentions, and negative outcomes can decrease productivity by undermining cooperative behavior just as moral behaviors, positive intentions, and positive outcomes can increase the value of human capital by supporting human cooperation.

### 5.3. The deed differentially affects behavioral willingness *via* moral judgment depending on Agent and Consequences

We found evidence for a moderated mediation effect, namely that the effect of the Deed on willingness to cooperate was partially mediated *via* moral judgments (supporting Hypothesis 7). The indirect effects of the Deed *via* moral judgments, however, depend on the valence of the Agent and the Consequences. These indirect effects are weakest when both Agent and Consequences have a negative valence, and they are strongest when both Agent and Consequences have a positive valence (see Hypotheses 8 and 9). This suggests that congruence between different subcomponents of moral judgment may have synergistic effects. Thus, given that moral judgment and, consequently, behavioral willingness are affected by the three components, interventions (e.g., developing codes of ethics) building up on this should be particularly effective (DiFonzo et al., 2020). For example, organizations may strive to engage in morally exemplary activities (i.e., Agent+, Deed+, Consequence+) in order to capitalize on the positive effects.

### 5.4. Direct conditional effects of the Deed on behavioral willingness

In addition to the conditional indirect effects of the Deed, we also found conditional remaining direct effects. That is, besides the mediation process *via* moral judgments, the Deed has a conditional direct effect on willingness to cooperate which is strongest when both Agent and Consequences have a positive valence (supporting Hypotheses 10 and 11). The conditional direct Deed effects appeared to be smaller if one of the other components was negative, and no conditional direct Deed effect was found when both other components were negative. Reasons for such remaining effects on higher willingness to cooperate could be that positive Deeds, especially if coupled with positive Agent intentions or positive Consequences (Robinson, 2018), increase trust in the cooperation partner and/or in the likelihood of positive personal monetary compensation (given higher productivity of the team work). This may imply that ethical training in organizations needs to be tailored to increase Deed-specific ethical prototypes and norms, cultivate virtues, and detect the bad consequences as a means of reinforcing all three relevant moral aspects. There is some evidence supporting our assertion: Research by Sturm (2017) highlights the importance of ethical prototypes (moral judgments triggered by the mere presence of stimuli rather than deliberate thought, which are accurate if they match widely accepted moral norms) and moral awareness in training procedures for management. Kim and Loewenstein found that limited knowledge of an ethical principle is one source of failure to make moral decisions and that this effect could be overcome by analogical teaching methods explicitly informing workers of ethical principles. Such education increased employees' likelihood to display spontaneous moral awareness and to make an ethical decision (Kim and Loewenstein, 2021). In terms of cultivating virtues, Chen et al. (2019) note that in order to boost organizational commitment, training courses should be offered to improve the moral virtues of the supervisor and to guide them to act in an ethical manner. This relates to other work that emphasizes how explicit knowledge and training of (plural) ethical theories and principles can lead to increased moral behavior (Shawver and Miller, 2017; van Gils et al., 2017). Therefore, the ADC Model is a promising alternative for holistic organizational ethics approaches. More work needs to be done to specifically test the effects of such (ADC Model based) interventions.

### 5.5. Strength, limitations, and directions for future research

One strength of our study is the use of a large nationwide sample of employed adults. This may allow for better generalizability in comparison to student or crowd-sourced samples. The experimental design has the advantage of avoiding self- or other- selection of individuals in certain situations and thus allows for a more causal test of assumptions.

Measuring behavioral willingness is not the same as observing behavior (Grasmick and Bursik, 1990; Exum and Bouffard, 2010; Petzold and Wolbring, 2018; Eifler and Petzold, 2019). However, studies reported substantial correlations between willingness measures and behavior (Beck and Ajzen, 1991; Pogarsky, 2004). They also found similar treatment effects in factorial surveys when comparing them to other designs (Hainmueller et al., 2015; Petzold and Wolbring, 2018). Still, replication with behavioral outcomes would be beneficial; however, large sample sizes would be needed in lab-settings to examine the complex interaction pattern tested here. Moreover, future studies may need to examine our findings in other (cultural, linguistic, and organizational) contexts to understand their degree of generalizability.

## 6. Conclusion

We set out to explore how a recently proposed model of moral judgment, the ADC Model, accounts for three specific conditions of moral judgment and the interaction of these conditions as well as whether these conditions and their interaction affect the willingness to cooperate in the workplace through moral judgment. We investigated the important problem of drug misuse in the workplace and examined whether the Agent, Deed, and Consequence components mapped to important moral considerations which would explain moral judgments and willingness to cooperate. The ADC Model not only explained how moral judgment can be envisioned as a three-dimensional process in which each component is expected to play a role, but especially that a congruence of all dimensions (either positive or negative) leads to strong positive or negative judgments. The results suggest that the Deed component plays a central role in these judgments, while the Agent and Consequence components moderate the Deed effects—whereby a congruence of Agent and Consequence with the valence of the Deed leads to a reinforcement of this effect. Moreover, our results also suggest that moral judgments are impactful, i.e., they are a strong mediator of the effects of the Deed on willingness to cooperate, while the Agent and the Consequences moderate this process. Although the Deed component appears to have particularly marked effects, organizational ethics interventions may especially benefit from building on the robust mutually reinforcing effects of positive Agent, Deed, and Consequences in alignment to promote moral behavior and signal very clearly immoral behavior. Inconsistent alignment of the three components may undermine moral integrity and holistic integration of the dimensions of human morality.

## Data availability statement

The raw data supporting the conclusions of this article are available here: Sattler et al. (2022). Any further enquiries can be directed to the corresponding author, SS, sebastian.sattler@uni-bielefeld.de.

## Ethics statement

The studies involving human participants were reviewed and approved by Ethics Committee of the University of Erfurt (reference number: EV-20190917). The patients/participants provided their written informed consent to participate in this study.

## Author contributions

SS: conceptualization, methodology, investigation, statistical analysis, data curation, writing—original draft, visualization, project administration, and funding acquisition. VD and ER: conceptualization and writing—original draft. All authors read and approved the final manuscript.
